# Specialized pediatric palliative care in Italy: where are we going? The Palliped 2022–2023 study

**DOI:** 10.1186/s13052-025-01850-x

**Published:** 2025-01-25

**Authors:** Franca Benini, Anna Mercante, Sara Di Nunzio, Simonetta Papa, the PalliPed 2022-2023 Working Group, Sergio Amarri, Laura Barrocu, Marco Bolognani, Monica Calì, Gaetano Catalano, Loredana Celentano, Lucia De Zen, Pierina Lazzarin, Stefano Lijoi, Luca Manfredini, Grazia Molinaro, Paola Moliterni, Rocco Orofino, Federico Pellegatta, Simone Pizzi, Marina Raspi, Michele Salata, Assunta Tornesello, Cesare Vezzoli

**Affiliations:** 1 Pediatric Palliative Care, Pain Service, Department of Women’s and Children’s Health, University Hospital of Padua, Padua, Italy; 2 Department of Biomedical and Neuromotor Sciences (DIBINEM), University of Bologna, Bologna, Italy; 3 Polistudium SRL, Milan, Italy

**Keywords:** Pediatric palliative care, Life-limiting conditions, Life-threatening conditions, Terminal illness, National network

## Abstract

**Background:**

The PalliPed project is a nationwide, observational, cross-sectional study designed with the aim of providing a constantly updated national database for the census and monitoring of specialized pediatric palliative care (PPC) activities in Italy. This paper presents the results of the first monitoring phase of the PalliPed project, which was developed through the PalliPed 2022–2023 study, to update current knowledge on the provision of specialized PPC services in Italy.

**Methods:**

Italian specialized PPC centers/facilities were invited to participate and asked to complete a self-reporting, ad-hoc, online survey regarding their clinical activity in 2022–2023, in the revision of the data initially collected in the first PalliPed study of 2021.

**Results:**

18 specialized PPC centers/facilities from 14 Italian regions and two autonomous provinces participated; 13 were identified as regional referral centers (72.2%), with the acquisition of three new centers in comparison to 2021. Full coverage of the regional territory was reported by 54% of them, compared with 45% in 2021, while a 24/7 service was offered by 23%, compared with 27% in 2021. Eight of 13 referral centers (61%) had a dedicated team, compared with 91% in 2021. Also, an overall increase in the number of followed patients was observed, rising from 1,209 (2019) to 2,734 (2023). In line with previous data, most PPC healthcare providers were nurses (*n* = 181) and physicians (*n* = 89), with an overall increased number of PPC providers from 2021.

**Conclusions:**

The nationwide PalliPed project established the first comprehensive overview and monitoring of the state of specialized PPC in Italy. Data reported within the PalliPed 2022–2023 monitoring study suggest a general improving trend in the availability of the specialized PPC service in Italy, compared with data collected in 2021. At the same time, the need for a greater effort to provide better care models and resources for specialized PPC remains, especially considering that the number of children needing PPC is constantly increasing.

**Supplementary Information:**

The online version contains supplementary material available at 10.1186/s13052-025-01850-x.

## Introduction

The prevalence of children with severe disabilities and/or incurable diseases has increased markedly in the last decades [1]. Indeed, medical and technological advances have prolonged the survival of patients, reducing the mortality associated with these conditions and generating, at the same time, new care needs that specialized pediatric palliative care (PPC) are called to address [2].

With regard to the Italian scenario, it has been estimated that 20,540–32,864 children and adolescents require PPC and that 18/100,000 inhabitants (all ages) require a specialized PPC service, defined as a dedicated setting including an interdisciplinary team of experts in PPC [2, 3]. To respond to this growing need, it is necessary to enhance the accessibility and quality of specialized Italian PPC services, providing improvement plans based on accurate data and precise definitions of existing gaps.

In line with this need, the Italian PPC network is organized into regional referral centers and other specialized facilities aimed at ensuring equitable access to palliative care for children with complex needs, as mandated by national healthcare policies (law 38/2010). Within this context, available informations on the actual implementation of the PPC network in Italy is scant, and this gap hampers the organization of PPC by Italian policymakers.

To fill this gap, the nationwide, observational, cross-sectional PalliPed project was designed to provide a constantly updated national database for the census and monitoring of specialized PPC activities in Italy. In the first part of the project, developed through the PalliPed 2021 study, information on the PPC burden and the quality and extent of specialized PPC networks/facilities in Italy were collected through the provision of two ad hoc surveys, providing for the first comprehensive demographic and clinical database of patients requiring specialized PPC in Italy [4, 5]. Collected data showed an inadequate extension of the PPC service, which resulted in covering only 15% of the estimated portion of pediatric patients who require specialized PPC in Italy [4]. Most of the patients were between 6 and 16 years old (45%), and the number of patients < 1 month was not consistent with the reported rate of deaths occurring before 28 days of age, suggesting the lack of a prompt referral to PPC by neonatologists. The necessity for more family support measures also emerged, particularly for mothers at work and social levels [4]. With regard to specialized PPC provision, 19 specialized centers/facilities provided their data concerning the extent and organization of service as of 2021 [5]. Among them, 11 were regional referral centers, with a dedicated team in 10 cases; less than half (45%) of centers covered the entire regional territory, and three offered 24/7 service. Seven Italian regions, mainly in central-southern Italy, reported no PPC centers/facilities. The dedicated staff resulted to be inadequate, and most healthcare providers were not recognized at an institutional level [5].

The regular monitoring of activities and data updates are essential to carry forward the PalliPed project and support the definition of proper healthcare interventions in specialized PPC. According to this aim, this paper presents the results of the PalliPed 2022–2023 study concerning the monitoring of the extent and organization of specialized PPC services in Italy up to 2023, along with a comparison with 2021 data to assess the evolution of the specialized PPC provision.

## Methods

### Project overview

PalliPed was designed in 2021 as an observational cross-sectional study involving all the Italian centers/facilities providing specialized PPC (see [4, 5] for full details on study design). The project was coordinated by The Pediatric Pain and Palliative Care Service of Padua University with the support of Fondazione Maruzza Lefebvre D’Ovidio Onlus.

Within the PalliPed 2022–2023 study, participating centers were asked to complete a self-reporting, ad-hoc, online survey considering the 2022–2023 activity period. The informations were retrieved by administrative database of centers. The survey was sent in January 2024 and closed in May 2024. The PalliPed 2022–2023 study was conducted in accordance with the Declaration of Helsinki, and the protocol was approved by the Ethics Committees of all participating centers. All the participants ≥ 18 years or legal guardians for younger children gave their consent to the use of medical records for research purposes.

### Survey structure

The PalliPed 2022–2023 survey consisted of 20 questions, divided into three areas: (1) general information on the PPC center/facility and characteristics of the service provided, such as geographical region, territorial coverage, 24/7 availability, presence of a dedicated team, number of beds, organizational changes occurred in the study period (16 items); (2) clinical activity data about the study period (two items); and (3) number of healthcare providers working in or with the facility, expressed as the total number and full-time equivalent (FTE; one FTE was intended as a person working full time, e.g., two part-time workers correspond to one FTE) up to the 31 December 2023 (two items). With regard to organizational changes, clinical activity and healthcare providers, data from the 2021 survey were retrieved to provide for a comparison with the 2022–2023 period data.

The full text of the survey and study definitions are available as supplementary material (Appendix I).

### Statistical analysis

All data were analyzed using descriptive statistics. The SPSS vs. 2214 software was used for data analysis.

## Results

### General information

Overall, 18 specialized PPC centers/facilities from 14 Italian regions (Basilicata, Campania, Emilia-Romagna, Friuli, Lazio, Liguria, Lombardy, Marche, Piedmont, Puglia, Sicily, Tuscany, Valle d’Aosta, Veneto) and two autonomous provinces (Trento and Bolzano) participated in the survey. Compared to 2021 data, two additional regions were represented in this study (Marche, Valle d’Aosta), while for both Emilia-Romagna and Friuli Venezia Giulia, one of the two centers reported in 2021 entered the regional network and works in coordination with the regional referral center. No specialized PPC centers/facilities were present in the remaining five Italian regions (Abruzzo, Calabria, Molise, Sardegna, Umbria). Details on the distribution of the 18 participating centers among Italian regions and specific provided services are reported in Table 1 and *Supplementary Table 1*, respectively.

**Table 1:** Distribution of the 18 participating centers among Italian regions and type of service

Regions	Centers/facilities, *n* (%)
Basilicata^a^	1 (5.3%)
Campania^a^	1 (5.3%)
Emilia-Romagna^a^	1 (5.3%)
Friuli Venezia Giulia^a^	1 (5.3%)
Lazio^a^	1 (5.3%)
Liguria^a^	1 (5.3%)
Lombardy	2 (10.5%)
Marche^a^	1 (5.3%)
Piedmont^a^ and Valle D’Aosta^b^	1 (5.3%)
Autonomous province of Trento^a^	1 (5.3%)
Puglia^a^	2 (10.5%)
Sicily	2 (10.5%)
Tuscany^a^	1 (5.3%)
Autonomous province of Bolzano^a^	1 (5.3%)
Veneto^a^	1 (5.3%)

^a^Regional Referral Center

^b^PPC activities referred to the Piedmont regional referral center

Among participating centers, 15 (83.3%) were within the national healthcare system (NHS), 3 (16.6%) were accredited as private entities, and 1 (5.5%) was a non-profit organization.

Regional referral centers (please see Appendix I for definitions) were 13 (72.2%), namely Basilicata, Campania, Emilia-Romagna, Friuli Venezia Giulia, Lazio, Liguria, Marche, Piedmont, Puglia, Tuscany, Veneto, the autonomous province of Trento of and of Bolzano. Among the five non-referral centers, four were in regions where a referral center was not present (Lombardy and Sicily), while the other (Puglia) collaborated with the regional referral center.

### Characteristics of service

#### Territorial coverage

Among the 13 regional referral centers, seven covered the entire regional territory (53.8%; Veneto, Liguria, Friuli Venezia Giulia, Piedmont, Emilia-Romagna, autonomous province of Bolzano, autonomous province of Trento). The other eleven (61.1%) participating centers reported partial territorial coverage.

#### Continuity of care

Three (23.1%) referral centers were reported to offer 24/7 service (Liguria, Puglia, Veneto), and 6 (46.1%) continuity of care for 8–20 h (Table 2). In the five non-referral centers, continuity of service was reported 24/7 by two facilities (40.0%; Lombardy, Sicily); in the other two centers (40.0%), it was provided for 8–20 h (Table 2).

**Table 2:** Continuity of care

	Referral centers (*n* = 13), n (%)	Other centers (*n* = 5), n (%)
24/7	3 (23.1)	2 (40.0)
8–20 h	6 (46.1)	2 (40.0)
8–16 h	1 (7.7)	1 (20.0)
8–18 h	1 (7.7)	–
Not active	1 (7.7)	–
Only on consultation	1 (7.7)	–

#### Dedicated team

Ten centers (55.5%) had a dedicated team. Among them, eight were referral centers (61.5% of referral centers). Five (27.7%) centers reported a mixed team composed of dedicated and non-dedicated staff (three were referral centers) (Table 3).

**Table 3:** Type of team

Type	*n* (%)
Dedicated team	10 (55.5%)
Mixed team	5 (27.7%)
Non-dedicated healthcare professionals	1 (5.5%)
Other	2 (11.1%)

#### Pain service

Eight (44,4%) centers reported specialized pain therapy (PT) activity at a regional level. In five cases, these PT centers were in the presence of a pediatric hospice and dedicated team.

#### Other data

The median of available beds was 4 (min–max: 1–20). Details on organizational changes are summarized in Table 4.

**Table 4:** Organizational changes in the period 2022–2023 and comparison with 2021 data

Type of change	2022–2023 data, *n* (%)	2021 data, *n* (%)	Delta values
Establishment of new regional referral center	13 (68.4%)	11 (57.8%)	+ 3 (10.6)^†^
Presence of a dedicated team (all centers)	10 (52.6%)	14 (73.6%)	−4 (21.0)
Extended hours of coverage to 24/7 (all centers)	5 (26.3%)	7 (36.8%)	−2 (10.5)
Extension to the full regional coverage area (regional referral centers)	9 (69.2%)	5 (45.4%)	+ 4 (23.8)
Pediatric Hospice Opening	8 (42.1%)	7 (36.8%)	+ 1 (5.3)
None	–	–	8 (42.1%)
Other changes	–	–	5 (26.3%)

^†^Three new referral centers were reported in the PalliPed 2022–2023 survey; PPC center present in Sicily was not reported as referral center in the PalliPed 2022–2023 survey

### Clinical activity in the period 2022–2023

**Fig. 1:**
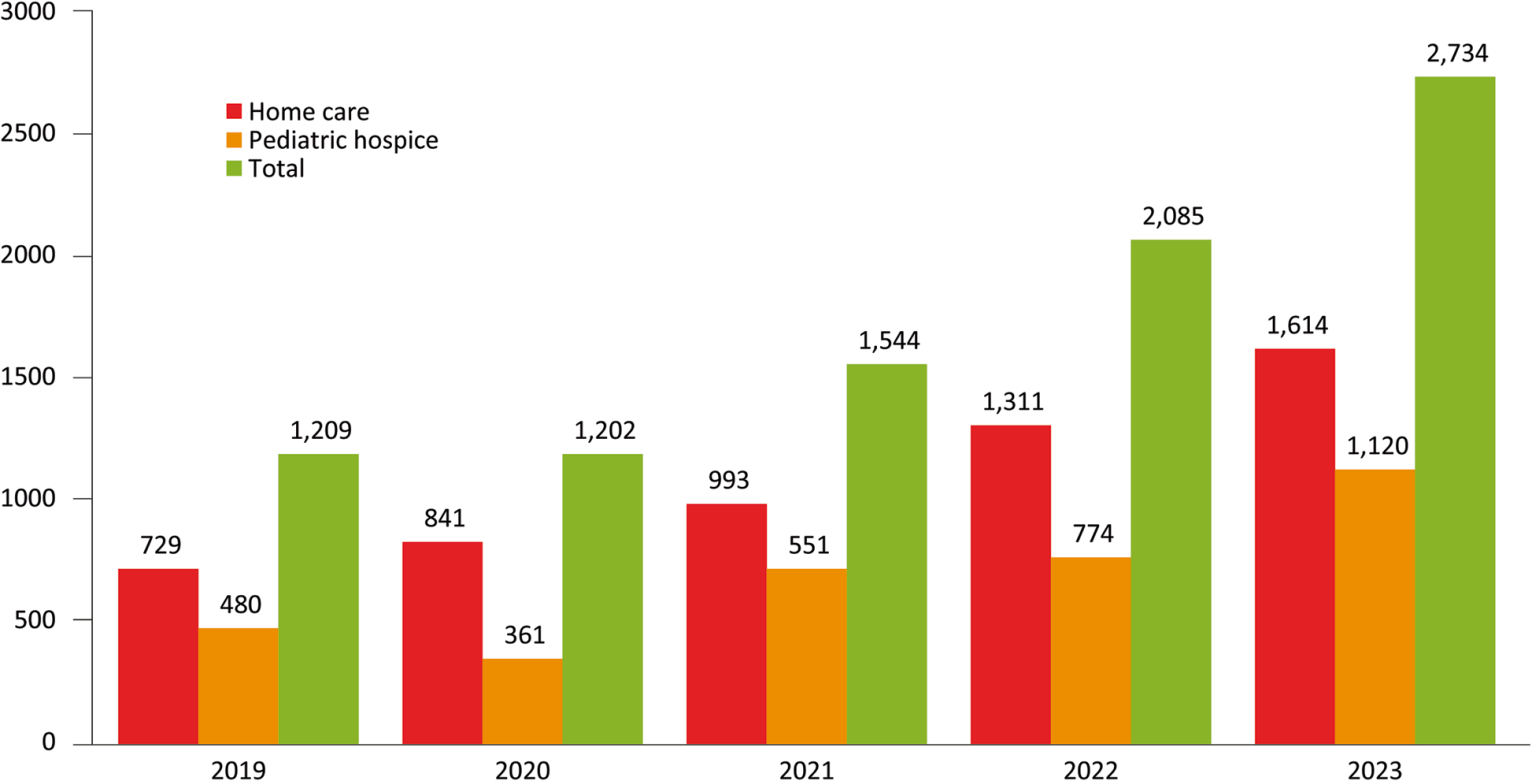
Comparison of activity data in the period 2019–2023

Table 5 summarizes the 2022–2023 clinical activity data. Overall, 2,085 patients (median per center: 66; range: 0–568) were followed in 2022 and 2,734 patients (median per center: 63; range: 0–693) in 2023 (Fig. 1). Patients in the care of the specialized PPC centers/network were 1,669 (median number of patients per center: 55; range: 0–634) as of 31 December 2022 and 1,906 (median number of patients per center: 57; range: 0–718) as of 31 December 2023. Overall, an increase in the number of patients was observed in the period between 2019 (*n* = 1,209) and 2023 (*n* = 2,734) (Fig. 1).

**Table 5:** Clinical activity data in the period 2022–2023

	**2022**	**2023**
Home care:		
• Total	1,311	1,614
• Median (range)	60 (0–493)	59.5 (0–588)
Pediatric hospice:		
• Total	774	1,120
• Median (range)	44.5 (0–285)	53 (0–346)
Total:		
• Total	2,085	2,734
• Median (range)	66 (0–568)	63 (0–693)

### Healthcare providers

**Fig. 2:**
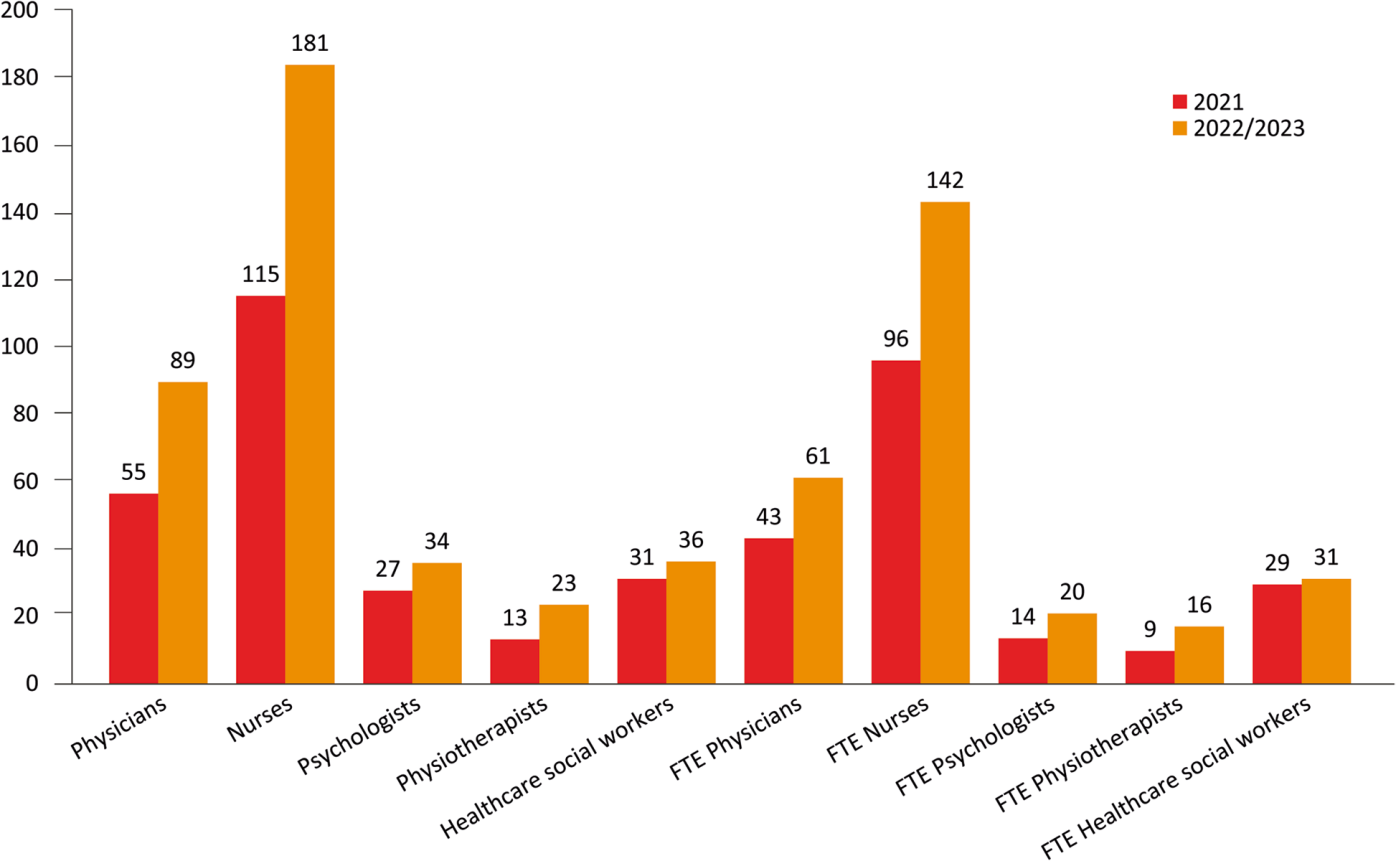
Comparison of the distribution of PPC healthcare providers in the period 2021–2023

Table 6 summarizes the distributions of PPC healthcare providers among all participating centers and the related FTE as of 31 December 2023. According to the FTE, most PPC healthcare providers were nurses (*n* = 181 providers; 142.5 FTE), followed by physicians (*n* = 89; 61.3 FTE) and healthcare social workers (*n* = 36; 31.0 FTE) (Table 6). Overall, an increase in the number of PPC healthcare providers was observed in the period between 2021 and 2023 (Fig. 2).

**Table 6:** Distributions of healthcare providers among participating centers

Healthcare providers	Total	Median	Full-time equivalent
Physicians	89	2	61.3
Nurses	181	5	142.5
Psychologists	34	1	20.1
Physiotherapists	23	0	16.0
Healthcare social workers	36	0	31.0
Administrative activity	14	0	6.75
Others	31	0	19.5

## Discussion

The Italian Ministry of Health started promoting the creation of regional and national networks of PPC and pain care centers in 2010 [6]. The coordination by at least one regional referral center was established as a requirement to ensure the continuity of care from hospital to home, as well as the definition of a multidisciplinary clinical pathway (law 38/2010; art. 2; art. 5) [6].

To enable the implementation of this organizational model and define resource allocation by the NHS, the regular assessment of the extent and organization of specialized PPC networks/facilities and the number of dedicated resources, as well as the characterization of this model of care, represent necessary steps.

PalliPed is the first nationwide project in Italy focused on collecting, describing and monitoring the characteristics of patients requiring specialized PPC, as well as the extent and structure of specialized PPC networks and facilities [4, 5].

Utilizing two self-reported online surveys, the project documented data on 867 patients and their families, along with the activities of 19 PPC centers as of 2021, offering the first comprehensive national assessment of specialized PPC services [4, 5].

This paper presents the results of the PalliPed 2022–2023 study, which represents an update on data regarding the organization and extent of specialized PPC networks and facilities up to 2023, along with a comparison with 2021 data.

In 2021, the 19 centers participating in the PalliPed project reported 11 regional referral centers. Seven Italian regions, mainly in central-southern Italy, reported no PPC centers/facilities [5]. The present update showed the addition of three regional referral centers (autonomous province of Bolzano, Marche and Puglia) in the period 2022–2023, while the PPC center present in Sicily was not reported as a referral center, bringing the overall number of referral centers from 11 to 13. The reduction from 7 to 5 of the Italian regions without PPC centers/facilities was also reported.

Territorial coverage of specialized PPC service, continuity of care and the availability of a dedicated team were partially maintained in the last years, compared with 2021 data [5]. In detail, the full coverage of regional territory was reported by 53.8% of regional referral centers, compared with 45.4% in 2021 [5]. The 24/7 service was reported by the 23.1% of referral centers and was 27.0% in 2021. Eight referral centers out of 13 (61.5%) had a dedicated team, compared with 10 (91.0%) centers out of 11 in 2021 [5]. Eight centers (44%) were regional PT centers, with pediatric hospice in 5 (28%) cases. Organizational changes reported in the period 2022–2023 were mainly the establishment of new referral centers (*n* = 3; 11%), the extension to the full regional coverage area (*n* = 4; 24%) and the opening of a new pediatric hospice.

An overall increase in the number of followed patients was observed in the period 2019–2023, rising from 1,209 (2019) to 2,734 (2023). Considering the 2021 estimates of children requiring specialized PPC in Italy (about 10,600) [3], current data suggest that the need for PPC was covered by 26% in 2023, reporting an 11% increase compared with 2021 [5]. Even if this percentage is still lower if reported to the current need for specialized PPC, available data suggests an upward trend and the improvement of PPC provision at a national level.

In line with previous data, most PPC healthcare providers were nurses (*n* = 181) and physicians (*n* = 89) [5]. If compared with 2021, the overall number of PPC providers increased, in particular, the number of nurses (+ 38%) and physicians (+ 62%), which were 131 and 55, respectively, in 2021 [5]. However, the number of PPC providers remained far from the real need for dedicated resources.

Overall, data reported within the PalliPed 2022–2023 study suggest an improvement trend in the extent of the specialized PPC service in Italy in the last few years. At the same time, the need for a greater effort to provide for a better reorganization of care models and improvement of resources in specialized PPC is still present, also considering that the number of children needing PPC is constantly increasing [7, 8]. Moreover, important critical issues persist, especially related to the variability and heterogeneity of the provision of specialized PPC in the different regions (both at an organizational level and in terms of the provision of dedicated resources). In particular, an inequality persists between northern and southern Italy (five regions that do not provide specialized PPC are all in southern Italy). Consequently, it will be important to work at an organizational level through the continuous monitoring of the extent of specialized PPC services available to children and families, as well as to work on the further implementation and training of healthcare providers. It will also be important to improve communication at a social level, which is necessary to implement awareness at a global level.

As previously reported, the design of the PalliPed study presents some limitations, such as the ad-hoc survey design and the self-reporting method of data collection. However, the PalliPed project establishes the first comprehensive overview of the current state of specialized PPC in Italy.

## Conclusions

By highlighting the areas for improvement, the study offers valuable insights for enhancing the quality and accessibility of specialized PPC services, representing a fundamental step to support the definition and implementation of the proper NHS supporting measures.

## Supplementary Information

Supplementary Material 1.

Supplementary Material 2.

## Data Availability

All data generated or analyzed in this study are included in this article and/or its figures. Further inquiries can be directed to the corresponding author.

## Abbreviations

FTE: Full-time equivalent
PPC: Pediatric palliative care
