# Supplementary material for: Specialized pediatric palliative care in Italy: where are we going? The Palliped 2022–2023 study
Source: Ital J Pediatr. 2025 Jan 25;51:15. doi: 10.1186/s13052-025-01850-x (PMC11763133; doi:10.1186/s13052-025-01850-x)
Supplement: Supplementary file 1 — Supplementary Material 1. [file 13052_2025_1850_MOESM1_ESM.docx]

**PALLIPED QUESTIONNAIRE 2022-2023**

**SPECIALIST PPCs**

**A1. Characteristics of the Network/Regional Reference Centre/Structure/Service***

*The questionnaire refers to the following definitions of Network/Regional Referral Centre/Paediatric Hospice/Paediatric Palliative Care Facility or Service/Specialist Paediatric Palliative Care Team

**Glossary**

| **Regional Network of Pediatric Pain Therapy and Palliative Care** |
| --- |
| The Regional Network of Pain Therapy (TD) and Paediatric Palliative Care (PPC) means a functional and integrated aggregation of pain therapy and palliative care activities aimed at minors, provided in the different care settings in a territorial and hospital area defined at the regional level (State-Regions Agreement of 25 July 2012, Rep. acts no. l5l/CSR). **The Network provides for the presence of a Reference Center, a dedicated Team and a pediatric Hospice. Coverage is on a regional or regional macro-area.** |

| **Regional Reference Centre for Paediatric Palliative Care and Pain Therapy** |
| --- |
| The Regional Reference Centre for Paediatric Palliative Care (PPC) and Pain Therapy (TD) refers to the clinical, organizational, training and research reference structure of the Network. The Centre is a public structure established by a formal act of the Region, dedicated and specific from an organizational and resource point of view for the pediatric age, with its Manager.  **This structure requires the presence of a trained team specifically dedicated to the management of minors in PPC at home and in the different life settings of the patient and his family. Coverage is on a regional or regional macro-area.** |

| **Residential Centre for Paediatric Palliatives (Paediatric Hospice)** |
| --- |
| A pediatric hospice is an alternative residential facility to the hospital, **dedicated and specific only to the pediatric patient and possibly to the young adult. It is a structure included within the regional network of TD and PPC,** with high care complexity, independent and autonomous from a logistical, organizational and management point of view. |

| **Specialist Paediatric Palliative Care Team (not Centre or Regional Network)** |
| --- |
| The specialist pediatric palliative care team at home is made up **of dedicated professionals with proven experience in PPC and pain therapy, as well as in highly complex specialist activities**. The team guarantees the specialist management of the PPC in interaction and integration with the disease specialist and all the other actors in the Network, providing hospital and home services. |

| **Paediatric Palliative Care Service/Team** |
| --- |
| The **palliative care team** is not autonomous from an organizational point of view but integrated within other services (e.g., Pediatrics, Anesthesia, Community Pediatrics or territorial services), which by company mandate follows with total or partial dedicated time children in PPC in hospitals and/or at home. |

| **A1.1 Autonomous Region/Province** |  |
| --- | --- |
| **A1.2.1 Type of service (fill in according to the definitions above)**  *One answer* | *□* **Regional Network** of Pain Therapy and Paediatric Palliative Care (presence of a Reference Centre, Paediatric Hospice and dedicated Team)  *□*  **Regional Network** of Pain Therapy and Paediatric Palliative Care without Paediatric Hospice (presence of Reference Centre and dedicated Team)  *□*  **Regional Reference Centre** for Paediatric Palliative Care and Pain Therapy with hospital but not home activity)  *□*  **Regional Reference Centre** for Paediatric Palliative Care and Pain Therapy with hospital and home activities in the Service Network  *□*  **Residential** Pediatric Palliative Center (Pediatric Hospice) only  *□* **Specialist Palliative Care Service/Team (not a Reference Centre or TD and PPC Network)** with dedicated and institutionally recognized staff who operate at home and/or hospital level, integrating or not with other links of the possible regional Network  *□* **Residential** Center for Pediatric Palliatives (Pediatric Hospice) with **home activities**  *□*  **Specialist Paediatric Palliative Care Team**, which is not autonomous from an organizational point of view and integrated within other services which, by company mandate, is PPC  *□* **Other** (specify) _____________________________________________________________________ |
| **A1.2.2 Name of the Network/Regional Reference Centre/Structure/Service** |  |
| **A1.3. Address** |  |
| **A1.4. Telephone number of the facility** |  |
| **A1.5. Accommodation e-mail address** |  |
| **A1.6. Compiler** | *Name* |
|  | *Surname* |
|  | *Profession* |
| **A1.7. Compiler telephone number** |  |
| **A1.8. Compiler email delivery** |  |
| **A1.9. Date of compilation** |  |
| **A1.10. The Regional Reference Centre/Paediatric Hospice/Paediatric Palliative Care Team is** | *□ Public Institution*  **□** *Accredited private body*  **□** *Other (please specify)* |
| **A1.11. Is the body you are presenting the Regional Reference Center for PPC and TD? (see definition)** | *□ Yes □ NO* |
| **A1.11.1 If not, does it collaborate with the Regional Reference Centre for PPC and TD?** | *□ NO*  *□ In our region, there is no Reference Center for Td and PPC deliberated*  *□ In our region, there is no Reference Center for TD and PPC* |
| **A1.12. What types of activities do you carry out?**  *As many answers as possible* | *□ Taking charge of PPC at home/affiliated residential facilities*  *□ PPC Consulting at Home*  *□ In Hospital, PPC Outpatient Clinic*  *□ In Hospital, PPC Admission*  *□ In hospital, PPC consulting activities*  *□ In hospital day hospital PPC*  *□ In pediatric hospice ordinary hospitalization PPC*  *□ In pediatric hospice day hospice PPC*  *□ In pediatric hospice PPC outpatient clinic*  *□ Consultancy activities in extra-regional specialist PPCs*  *□ In hospital, outpatient clinic, Specialist pain therapy*  *□ In hospital, hospitalization (ordinary/DH) Specialized pain therapy*  *□ In hospital, consultancy activities, Specialist pain therapy*  *□ Continuous availability for All the links of the Regional Network for Pain Therapy*  *□ Academic Training*  *□ Non-academic training*  *□ Research*  *□ Other (please specify)* |
| **A1.13. Continuity of home care active in taking charge for a 24-hour slot, 7 days a week** | *□ Yes □ NO* |
| **A1.13.1If no, please specify the time slot for continuity of home care** | *From /_________/ to /________/* |
| **A1.14. Territorial coverage** | *□ Covers the entire regional territory (or Provincial, for the Autonomous Provinces)*  *□ Covers only some areas of the regional territory. Specify areas of competence ___________________________________* |
| **A1.15. Team** | *□ The activity is carried out by a specialized multidisciplinary team of* ***dedicated and structured***  *professionals (i.e., belonging to simple or complex PPC and TD structures) who follow patients in different care settings (home and/or pediatric hospice and/or hospital)*  *□ The activity is carried out by a multidisciplinary specialist team of professionals made up of dedicated and structured professionals (i.e., belonging to simple or complex PPC structures) and professionals working in other organizational structures (e.g., adult palliative care, family pediatrics...), who intervene in the continuous and institutionally structured management of eligible subjects, with part-time work.*  *□ The activity is carried out by* ***non-dedicated professionals*** *who also work in different structures/services and who intervene in the management of eligible patients in individual situations and as needed through a non-institutionalized activity at an organizational level*  *□ Other specify _________________________________________________* |
| **A1.16. Number of currently active Paediatric Hospice beds** | *□ Number:*  *□ Not applicable* |
| **A1.17 Organisational changes that took place in the two-year period 2022 - 2023** | *□ Establishment by resolution of the Reference Centre*  *□ Institution with a resolution of the dedicated Team*  *□Extension of coverage hours*  *□Expansion of the coverage area*  *□ Opening of the Pediatric Hospice*  *□ Approval of the construction of the paediatric hospice*  *□ No organizational changes*  *□Other (specify)* |

**A2.** Activity data

| **A2.1. Indicate 2022 activity data** | *Total number of patients in home care in PPC in 2022 (in charge + deceased + discharged):*  *Total number of patients admitted to HP in 2022:*  *Number of patients in charge of the PPC Network/Service on 31/12/2022*  *□ Notes (please specify)* |
| --- | --- |
| **A2.2. 2023 activity data** | *Total number of patients in charge in PPC at home in 2023 (in charge + deceased + discharged):*  *Total number of patients admitted to HP in 2023:*  *Number of patients in charge of the PPC Network/Service on 31/12/2023*  *□ Notes (please specify)* |

**A3.** Healthcare Personnel

| **A3.1 Healthcare personnel working in the Regional Reference Network/Centre/Paediatric Hospice/Specialist Paediatric Palliative Care Team as of 31 December 2023** | *Total number of working staff:*  *Of which (indicate the number of operators):*   - *Physicians:* - *Nurses:* - *Psychologists:* - *Physiotherapists:* - *Social and health workers:* - *Administrative activity:* - *Other operators:* |
| --- | --- |
| **A3.2. FTE of dedicated healthcare workers as of 31 December 2023**  **FTE (Full Time Equivalent)** is equivalent to one person working full-time (regardless of the type of employment contract) **(e.g., two part-time at 50% correspond to 1 total unit)** | *Doctors (indicate the number of FTEs):*  *Nurses (indicate the number /FTE):*  *Psychologists (indicate the number of FTEs):*  *Physiotherapists (indicate the number of FTEs):*  *Social and Health Workers (indicate the number of FTEs):*  *Administrative activity (indicate the number of FTEs):*  *Other operators (indicate the number of FTEs) and specify the role:* |
