# Supplementary material for: Specialized pediatric palliative care in Italy: where are we going? The Palliped 2022–2023 study
Source: Ital J Pediatr. 2025 Jan 25;51:15. doi: 10.1186/s13052-025-01850-x (PMC11763133; doi:10.1186/s13052-025-01850-x)
Supplement: Supplementary file 2 — Supplementary Material 2. [file 13052_2025_1850_MOESM2_ESM.docx]

| **Supplementary Table 1. PPC Activities provided by regions** | |
| --- | --- |
| **Activities** | **Region** |
| Home care  Advice at home  Outpatient clinic in hospital  Hospitalization  Consultancy activities in hospitals  Day hospital  Hospitalization in hospice  Day hospital in hospice  outpatient clinic in hospice  Consultancy activities in extra-regional specialist  Outpatient clinic Specialist pain therapy  Hospitalization (ordinary/DH) Specialist pain therapy  Specialist pain therapy consultancy  Continuous availability for all the rings of the regional TD network  Academic training  Non-academic training  Research | Veneto |
| PPC advice at home  PPC outpatient clinic in hospital  PPC hospitalization  PPC consultancy activities in hospitals  Day Hospital PPC in Hospital  PPC hospitalization in hospice  Day Hospital PPC in hospice  PPC outpatient clinic in hospice  Outpatient clinic, specialist pain therapy  Specialist pain therapy consultancy  Academic training  Non-academic training  Other | Campania |

**Specialized Pediatric palliative care in Italy: where are we going? The Palliped 2022-2023 Study**

**SUPPLEMENTARY MATERIAL**

| PPC at home  PPC advice at home  PPC outpatient clinic in hospital  PPC hospitalization  PPC consultancy activities in hospitals  Day hospital PPC in the hospital  PPC hospitalization in hospice  Outpatient clinic, specialist pain therapy  Specialist pain therapy consultancy  Continuous availability for all the rings of the regional TD network  Academic training  Research | Friuli Venezia Giulia |
| --- | --- |
| PPC at home  PPC advice at home  PPC hospitalization  PPC consultancy activities in hospitals  PPC hospitalization in hospice  Consultancy activities in extra-regional specialist PPCs  Hospitalization (ordinary/DH) Specialist pain therapy  Specialist pain therapy consultancy  Continuous availability for all the rings of the regional TD network  Academic training  Non-academic training  Research | Liguria |
| PPC at home  PPC advice at home  PPC outpatient clinic in hospital  PPC hospitalization  PPC consultancy activities in hospitals  Day Hospital PPC in Hospital  Outpatient clinic, specialist pain therapy  Hospitalization (ordinary/DH) Specialist pain therapy  Specialist pain therapy consultancy  Non-academic training  Other | Trentino Alto Adige |
| PPC at home  PPC advice at home  PPC outpatient clinic in hospital  PPC consultancy activities in hospitals  Hospitalization (ordinary/DH) Specialist pain therapy  Specialist pain therapy consultancy  Continuous availability for all the rings of the regional TD network  Academic training  Non-academic training  Research  Other | Tuscany |
| PPC at home  PPC advice at home  PPC consultancy activities in hospitals  PPC hospitalization in hospice  Day Hospital PPC in hospice  PPC outpatient clinic in hospice  Academic training  Non-academic training  Research  Other | Lombardy (Milan) |
| PPC at home  PPC advice at home  PPC outpatient clinic in hospital  PPC hospitalization  PPC consultancy activities in hospitals  Day hospital PPC in hospital  Academic training  Non-academic training  Research | Emilia-Romagna |
| PPC consultancy activities in hospitals  PPC hospitalization in hospice  Day Hospital PPC in hospice  Consultancy activities in extra-regional specialist PPCs  Specialist pain therapy consultancy  Academic training  Non-academic training  Research | Latium |
| PPC outpatient clinic in hospital  PPC hospitalization  PPC consultancy activities in hospitals  Day Hospital PPC in Hospital  Academic training  Non-academic training  Other | Piedmont (pneumology) |
| PPC at home  PPC consultancy activities in hospitals  Specialist pain therapy consultancy  Continuous availability for all the rings of the regional TD network  Academic training  Non-academic training  Research | Autonomous Province of Trento |
| PPC advice at home  PPC outpatient clinic in hospital  PPC hospitalization  PPC consultancy activities in hospitals  Specialist pain therapy consultancy  Non-academic training | Marches |
| PPC advice at home  PPC hospitalization  PPC consultancy activities in hospitals  Specialist pain therapy consultancy  Non-academic training | Apulia (Lecce) |
| PPC advice at home  PPC outpatient clinic in hospital  PPC consultancy activities in hospitals  Outpatient clinic, specialist pain therapy | Lombardy (Brescia) |
| PPC hospitalization in hospice  Day Hospital PPC in hospice  PPC outpatient clinic in hospice | Sicily (Catania) |
| PPC at home  PPC advice at home  Academic training | Apulia (Bari) |
| PPC at home  Non-academic training | Sicily (Palermo) |
| PPC at home  Pediatric Hospice  PPC outpatient clinic in hospice | Basilicata |
